# Vpx rescue of HIV-1 from the antiviral state is independent of the intracellular nucleotide concentration

**DOI:** 10.1186/1742-4690-10-S1-P71

**Published:** 2013-09-19

**Authors:** Christian Reinhard, Dario Botinelli, Baek Kim, Jeremy Luban

**Affiliations:** 1 University of Geneva, Geneva, Switzerland; 2 Emory School of Medicine, Atlanta, USA; 3 University of Massachusetts Medical School, Worcester, USA

## Background

The SIV_MAC_/HIV-2 accessory protein Vpx recruits the CUL4A-DCAF1 E3 ubiquitin ligase complex to degrade the deoxynucleotide hydrolase SAMHD1. In cells that have low deoxynucleotide levels, such as myeloid cells and resting T-cells, this results in increased deoxynucleotides available for reverse transcription inhibiting a range of retroviruses. In this way, pretreatment of monocyte-derived dendritic cells (MDDC) with SIV_MAC_ virus-like particles containing Vpx (Vpx-VLP) enhances HIV-1 transduction. Vpx-VLP also rescue HIV-1, but not SIV_MAC_, from a type I interferon induced antiviral state (*Retrovirology* 2011, **8**:49) data suggest that SAMHD1 is dephosphorylated in non-cycling and IFN treated cells and in that dephosphorylated-SAMHD1 gains additional restriction capabilities via a yet unknown mode of action [1,2].

## Results

Here we show that, under conditions where HIV-1 transduction is rescued from the antiviral state in MDDC and SAMHD1 is degraded, Vpx-VLP did not increase intracellular deoxynucleotide levels. Both Vpx-VLP and exogenous nucleosides increased HIV-1 late reverse transcription products, but only Vpx-VLP increased the amount of 2-LTR circles and integrated proviruses in myeloid cells. Using exogenous nucleosides to achieve lentiviral vector transduction it was possible to transduce MDDC with expression vectors for Vpr and Vpx from different species without the use of Vpx VLP. We found that Vpr from SIV infecting mustached and De Brazza’s monkey was able to degrade SAMHD1 and increase HIV-1 cDNA in MDDC in the antiviral state.

## Conclusion

The data presented here provide evidence that prevention of deoxynucleotide hydrolysis by degrading SAMHD1 is not the only mode of action by which Vpx rescues HIV-1 from the antiviral state in MDDC. Vpx also relieves HIV-1 from a block in these cells that occurs after reverse transcription.
